# Antimicrobial resistance pattern, virulence determinants and molecular analysis of *Enterococcus faecium* isolated from children infections in Iran

**DOI:** 10.1186/s12866-019-1539-y

**Published:** 2019-07-08

**Authors:** Azin Sattari-Maraji, Fereshteh Jabalameli, Narges Node Farahani, Reza Beigverdi, Mohammad Emaneini

**Affiliations:** Department of Microbiology, School of Medicine, Tehran University of Medical Sciences, 100 Poursina St., Keshavarz Blvd, Tehran, Iran

**Keywords:** Enterococcus, Virulence factors, Antimicrobial resistance, MLVA

## Abstract

**Background:**

Enterococcus species continues to be an important cause of hospital-acquired infection worldwide. This study was designed to determine the antibiotic resistance profiles, virulence genes and molecular characteristics of *Enterococcus faecium* strains isolated from an Iranian children hospital in a four-years period.

**Results:**

A total 189 *Enterococcus* strains, comprising 108 (57%) *E. faecium*, 67 (35%) *E. faecalis* and 14 (7%) isolates of other spp. were isolated during the collection period. More than 92% of *E. faecium* isolates were resistant to ampicillin (92.5%), ciprofloxacin (96%), erythromycin (100%) and clindamycin (96%). A high frequency of resistance to clindamycin (100%), erythromycin (98.5%) and ciprofloxacin (80.5%) was observed among *E. faecalis* isolates, while resistance to ampicillin (7%) was less frequent. The prevalence of *vanA* gene among vancomycin resistant *E. faecium* and vancomycin resistant *E. faecalis* was 95 and 50%, respectively. The analysis of 108 *E. faecium* isolates revealed 34 variable number tandem repeat (VNTR) patterns and 27 Multi Locus VNTR Analysis (MLVA) types (MTs).

**Conclusions:**

The results show a shift from *E. faecalis* to *E. faecium* as the dominant enterococcal species among patients at the children Hospital. Our data revealed that the majority of *E. faecium* isolates (66%) belonged to three common MTs and these types were isolated from different wards in children hospital.

## Background

Enterococcus continues to be an important cause of hospital-acquired infection worldwide [1]. Two species (*Enterococcus faecalis* and *Enterococcus faecium*) are responsible for the majority of enterococcal infections in humans and these species have become resistant to multiple antimicrobial agents such as vancomycin (vancomycin resistant enterococci; VRE), aminoglycosides (the high-level gentamicin resistant; HLGR), macrolides, and tetracyclines [2, 3]. The glycopeptide resistance in enterococci is mediated by nine (*vanA*, *vanB*, *vanC*, *vanD*, *vanE*, *vanG*, *vanL*, *vanM*, *vanN*) mobile gene clusters [4]. Among them, *vanA* genotype is the most common type of enterococcal vancomycin resistance in several countries [5]. The presence of *aac (6′)-Ie-aph(2″)-Ia* gene, which is carried on transposon is the main cause of HLGR emergence [6, 7]. In addition to the increasing antibiotic resistance, some virulence determinants described to be associated with pathogenesis in *E. faecium* including, collagen-binding adhesin of *E. faecium* (Acm), aggregation substance (Asa1), cytolysin (CylA), enterococcal surface protein (Esp), gelatinase (GelE) [4, 8]. Recently, several reports have described Multilocus variable-number of tandem repeat analysis based on PCR-amplification of variable number tandem repeat (VNTR) located on chromosome, is a suitable tool for learning the genetic relationships of important bacterial pathogens, including *E. faecium* [3, 9]. Despite the high incidence rate of resistant enterococci in Iran, especially VRE and HLGR [10, 11], there is limited information on enterococcal strains isolated from children infections. This study was designed to determine the antibiotic resistance profiles, virulence genes and the prevalence of different VNTR patterns among *E. faecium* strains isolated from an Iranian children hospital in a four-years period.

## Results

**Fig. 1:**
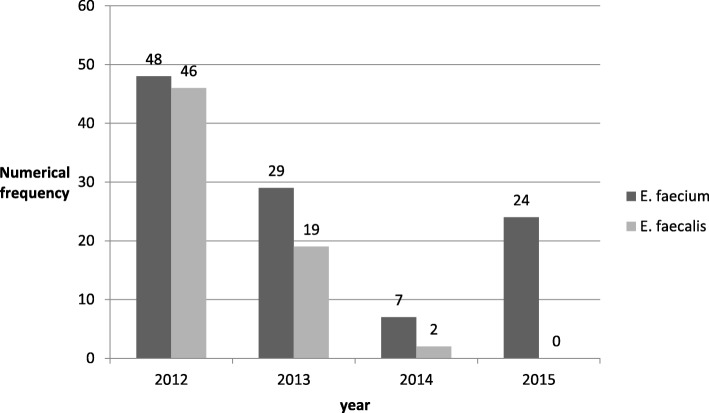
Distribution of *E. faecium* and *E. faecalis* isolates based on isolation time

A total 189 Enterococcus strains, comprising 108 (57%) *E. faecium*, 67 (35%) *E. faecalis* and 14 (7%) isolates of other spp. were isolated during the collection period. Distribution of *E. faecium* and *E. faecalis* isolates based on isolation time (Fig. 1) was showed that during 2015, the prevalence of *E. faecium* were significantly higher than *E. faecalis* (*P* = 0.0001). Most of the *E. faecium* strains (74%) were isolated from urine, followed by blood (11%), body fluids (7%) and wound (2%). The majority proportion of *E. faecium* isolates were obtained from urology hospitalized patients (14%) and outpatients (13%).

### Antimicrobial susceptibility

More than 92% of *E. faecium* isolates were resistant to ampicillin (92.5%), ciprofloxacin (96%), erythromycin (100%) and clindamycin (96%). A high frequency of resistance to clindamycin (100%), erythromycin (98.5%) and ciprofloxacin (80.5%) was observed among *E. faecalis* isolates, while resistance to ampicillin (7%) was less frequent. HLGR was found in 75 and 49% of *E. faecium* and *E. faecalis* strains‚ respectively. Inducible resistance to clindamycin was 7% among *E. faecium* isolates, but not in *E. faecalis* strains. Vancomycin resistance were detected in 70% of *E. faecium* and 9% of *E. faecalis* isolates. The MIC values of Vancomycin Resistant *E. faecium* (VREfm) and Vancomycin Resistant *E. faecalis* (VREfs) were ≥ 128 μg̸ml and ≥ 128 μg̸ml respectively. The prevalence of *vanA* gene among VREfm and VREfs isolates was 95 and 50%, respectively. The presence of *aac(6′)-Ie-aph(2″)-Ia* gene among HLGR isolates of *E. faecium* and *E. faecalis* was 48 and 67%, respectively.

### Prevalence of virulence genes among *E. faecium* isolates

The *acm* was the most commonly detected gene (81%), followed by *esp* (17.5%), *gelE* (16%), and *ace* (6%). Only two (2%) isolates carried *asa1* gene and *cylA* was not seen in any of the isolates. The presence of the *esp* gene was significantly higher (*P* = 0.011) among VREfm isolates than vancomycin sensitive *E. faecium* isolates.

### Molecular analysis of *E. faecium*

The results of MLVA typing of *E. faecium* isolates are presented in Table 1. The analysis of 108 *E. faecium* strains revealed 34 VNTR patterns and 27 MTs. Forty-three isolates (40%) were identified as MT1, 15 (13.8%) as MT2 and 14 (12.9%) as MT3. MT1 was isolated from different wards of the hospital, while MT2 and MT3 were not found in outpatients who were referred to this center. By comparing antibiotic resistance genes in three common types (MT1- MT3), *aac(6′)-Ie-aph(2“)-Ia* was significantly higher in MT3 than MT1 (*P* = 0.0046). Also, virulence gene *esp* had more frequency in MT3 than MT1 (*P* = 0.0003). The most prevalent pattern of antibiotic resistance in common types (MT1- MT3) was related to pattern gentamicin, ampicillin, ciprofloxacin, erythromycin, and clindamycin. Moreover, the results of the antibiotic resistance genes pattern in common types indicated that pattern *vanA*+ *aac(6’)-Ie-aph(2”)-Ia* in MT3 was significantly more frequent than MT1 (*P* = 0.013).

**Table 1:** Characteristics of *E. faecium* isolates

Ward^a^	Isolate	Time of isolation (m / y)^b^	Sample	Resistance pattern^c^	Resistance genes	Virulence genes	MLVA type (MT)
Out patient	1	1/2012	Urine	GM, AP, CIP, E, CD	*vanA*	*acm, esp*	1
	2	1/2012	Urine	AP, E, CD	*–*	*acm*	10
	3	6/2012	Urine	GM, AP, CIP, E, CD	*aac(6′)-Ie-aph(2″)-Ia*	*acm ،ace*	1
	4	6/2012	Urine	GM, AP, CIP, E, CD	*aac(6′)-Ie-aph(2″)-Ia*	*acm*	1
	5	6/2012	Urine	AP, CIP, E, CD^d^	*–*	*acm*	1
	6	6/2012	Urine	GM, AP, CIP, E, CD	*–*	*acm*	13
	7	9/2012	Urine	GM, AP, CIP, E, CD	*vanA*	*acm*	11
	8	10/2012	Urine	AP, CIP, E, CD	*–*	*–*	9
	9	6/2013	Urine	GM, AP, CIP, E, CD^d^	*–*	*–*	17
	10	7/2013	Urine	AP, CIP, E, CD	*vanA*	*acm*	1
	11	9/2013	Urine	AP, CIP, E, CD	*vanA*	*acm*	1
	12	9/2013	Blood	GM, AP, CIP, E, CD	*vanA, aac(6′)-Ie-aph(2″)-Ia*	*acm*	1
	13	11/2013	Urine	GM, AP, CIP, E, CD	*vanA*	*acm*	1
	14	4/2014	Urine	GM, AP, CIP, E, CD	*vanA*	*acm*	1
Urology	15	5/2012	Urine	GM, AP, CIP, E, CD	*vanA, aac(6′)-Ie-aph(2″)-Ia*	*acm*	20
	16	5/2012	Urine	GM, AP, CIP, E, CD^d^	*vanA*	*acm، ace*	25
	17	5/2012	Urine	AP, CIP, E, CD^d^	*–*	*acm، ace*	6
	18	6/2012	Urine	GM, AP, CIP, E, CD	*aac(6′)-Ie-aph(2″)-Ia*	*acm*	7
	19	6/2012	Urine	AP, CIP, E, CD	*–*	*–*	11
	20	6/2012	Urine	GM, AP, CIP, E, CD	*–*	*acm*	8
	21	7/2012	Urine	AP, CIP, E, CD	*–*	*–*	23
	22	8/2012	Urine	AP, CIP, E, CD	*–*	*–*	4
	23	9/2012	Urine	GM, AP, CIP, E, CD	*vanA, aac(6′)-Ie-aph(2″)-Ia*	*acm, esp*	3
	24	10/2012	Urine	GM, AP, CIP, E, CD	*–*	*acm, esp*	2
	25	6/2013	Urine	GM, AP, CIP, E, CD	*vanA, aac(6′)-Ie-aph(2″)-Ia*	*acm, gelE*	1
	26	11/2013	Urine	GM, AP, CIP, E, CD	*vanA*	*acm, esp*	3
	27	2/2015	Blood	GM, AP, CIP, E, CD	*vanA, aac(6′)-Ie-aph(2″)-Ia*	*acm*	2
	28	5/2015	Urine	GM, AP, CIP, E, CD	*vanA*	*acm*	3
	29	5/2015	Urine	GM, AP, CIP, E, CD	*vanA, aac(6′)-Ie-aph(2″)-Ia*	*acm, esp*	1
Surgery	30	5/2012	CSF	AP, CIP, E, CD	*vanA*	*acm*	1
	31	5/2012	CSF	AP, CIP, E, CD	*vanA*	*acm, esp*	1
	32	8/2012	Urine	AP, CIP, E, CD	*vanA*	*acm, esp*	1
	33	5/2013	Urine	CIP, E	*–*	*acm*	27
	34	6/2013	Urine	GM, AP, CIP, E, CD	*vanA*	*acm*	1
	35	11/2013	Urine	GM, CIP, E, CD	*aac(6′)-Ie-aph(2″)-Ia*	*asa1, gelE، ace*	5
	36	4/2014	Blood	GM, AP, CIP, E, CD	*vanA, aac(6′)-Ie-aph(2″)-Ia*	*acm, esp, gelE*	3
	37	1/2015	Urine	GM, AP, CIP, E, CD	*vanA*	*acm, esp*	2
	38	4/2015	Urine	AP, CIP, E, CD	*vanA*	*acm, gelE*	1
	39	12/2015	Urine	GM, AP, CIP, E, CD	*vanA*	*acm*	1
NICU	40	10/2012	Urine	GM, AP, CIP, E, CD	*–*	*–*	10
	41	8/2013	Tracheal aspirate	GM, AP, CIP, E, CD	*vanA*	*acm*	1
	42	1/2015	Urine	GM, AP, CIP, E, CD	*vanA*	*acm*	4
	43	1/2015	Urine	GM, AP, CIP, E, CD^d^	*vanA, aac(6′)-Ie-aph(2″)-Ia*	*acm*	1
	44	1/2015	Urine	GM, AP, CIP, E, CD	*vanA, aac(6′)-Ie-aph(2″)-Ia*	*acm*	2
	45	2/2015	Urine	GM, AP, CIP, E, CD	*vanA aac(6′)-Ie-aph(2″)-Ia*	*acm, esp*	2
	46	3/2015	Blood	GM, AP, CIP, E, CD	*vanA, aac(6′)-Ie-aph(2″)-Ia*	*acm, esp*	1
	47	8/2015	Blood	GM, AP, CIP, E, CD	*aac(6′)-Ie-aph(2″)-Ia*	*acm، ace*	1
	48	10/2015	Ascites	GM, AP, CIP, E, CD	*vanA*	*–*	2
	49	10/2015	Ascites	GM, AP, CIP, E, CD	*vanA, aac(6′)-Ie-aph(2″)-Ia*	*acm*	2
CICU	50	1/2012	Ascites	GM, AP, CIP, E, CD	*vanA*	*acm*	1
	51	9/2013	Urine	GM, AP, CIP, E, CD	*vanA*	*–*	1
	52	6/2013	Urine	CIP, E, CD	*vanA*	*acm,esp, gelE*	2
	53	1/2015	Urine	GM, AP, CIP, E, CD	*vanA, aac(6′)-Ie-aph(2″)-Ia*	*acm*	2
	54	3/2015	Ascites	GM, AP, CIP, E, CD	*–*	*–*	1
	55	5/2015	Urine	GM, AP, CIP, E, CD	*vanA*	*acm*	14
	56	6/2015	Blood	GM, AP, CIP, E, CD	*vanA, aac(6′)-Ie-aph(2″)-Ia*	*acm، ace*	3
	57	6/2015	Urine	GM, AP, CIP, E, CD	*vanA, aac(6′)-Ie-aph(2″)-Ia*	*acm، ace*	1
PICU	58	12/2012	Wound	GM, AP, CIP, E, CD	*vanA, aac(6′)-Ie-aph(2″)-Ia*	*acm, esp, gelE*	3
	59	4/2013	Tracheal aspirate	GM, AP, CIP, E, CD	*vanA, aac(6′)-Ie-aph(2″)-Ia*	*acm*	1
	60	1/2014	Wound	GM, AP, CIP, E, CD	*vanA*	*acm*	22
	61	3/2014	Blood	GM, AP, CIP, E, CD	*vanA*	*acm, esp*	2
	62	11/2014	Ascites	GM, AP, CIP, E, CD	*vanA*	*acm*	2
	63	2/2015	Urine	GM, AP, CIP, E, CD	*vanA*	*acm*	1
	64	3/2015	Blood	GM, AP, CIP, E, CD^d^	*vanA*	*acm*	2
	65	6/2015	Dialysis fluid	GM, AP, CIP, E	*vanA*	*acm*	1
Dialysis center	66	5/2012	Urine	AP, CIP, E, CD^d^	*–*	*acm*	1
	67	5/2012	Urine	GM, AP, CIP, E, CD	*vanA, aac(6′)-Ie-aph(2″)-Ia*	*acm*	1
	68	10/2012	Urine	E, CD	*–*	*asa1*	5
	69	1/2013	Catheter	GM, AP, CIP, E, CD	*vanA*	*acm, esp*	3
	70	1/2013	Urine	CIP, E, CD^d^	*–*	*–*	6
	71	6/2013	Urine	AP, CIP, E, CD	*–*	*acm*	1
	72	3/2014	Urine	GM, AP, CIP, E, CD	*vanA*	*acm, gelE*	4
Neonatal	73	12/2011	Urine	GM, AP, CIP, E, CD	*vanA*	*acm*	7
	74	5/2012		GM, AP, CIP, E, CD	*vanA*	*acm*	1
	75	6/2012	Urine	GM, AP, CIP, E, CD	*vanA, aac(6′)-Ie-aph(2″)-Ia*	*acm*	3
	76	9/2012	Urine	GM, AP, CIP, E, CD	*vanA, aac(6′)-Ie-aph(2″)-Ia*	*acm*	3
	77	9/2012	Urine	GM, AP, CIP, E, CD	*vanA*	*acm*	13
	78	12/2013	Urine	GM, AP, CIP, E, CD	*vanA*	*gelE*	18
Emeregency	79	1/2012	Urine	E, CD	*–*	*acm*	1
	80	1/2012	CSF	GM, AP, CIP, E	*aac(6′)-Ie-aph(2″)-Ia*	*acm*	1
	81	1/2012	Urine	GM, AP, CIP, E, CD	*–*	*acm*	19
	82	2/2013	Blood	GM, AP, CIP, E, CD	*vanA*	*acm, gelE*	1
	83	2/2013	Urine	GM, AP, CIP, E, CD	*vanA, aac(6′)-Ie-aph(2″)-Ia*	*acm, gelE*	1
	84	2/2013	Urine	GM, AP, CIP, E, CD	*vanA, aac(6′)-Ie-aph(2″)-Ia*	*acm, gelE*	2
Digestive	85	12/2011	Urine	GM, AP, CIP, E, CD	*–*	*–*	5
	86	10/2012	Urine	AP, CIP, E, CD	*–*	*–*	9
	87	1/2013	Blood	GM, AP, CIP, E, CD	*vanA, aac(6′)-Ie-aph(2″)-Ia*	*acm*	2
	88	2/2013	Urine	GM, AP, CIP, E, CD	*vanA, aac(6′)-Ie-aph(2″)-Ia*	*acm, gelE*	2
Rheumatology	89	2/2013	Urine	GM, AP, CIP, E, CD	*vanA, aac(6′)-Ie-aph(2″)-Ia*	*acm, esp, gelE*	3
	90	6/2013	Blood	GM, AP, CIP, E, CD	*–*	*acm*	1
	91	11/2015	sputum	GM, AP, CIP, E, CD	*vanA, aac(6′)-Ie-aph(2″)-Ia*	*acm, esp*	3
Neurology	92	3/2012	Urine	GM, AP, CIP, E, CD	*vanA*	*–*	21
Oncology	93	9/2015	Urine	GM, AP, CIP, E, CD	*vanA, aac(6′)-Ie-aph(2″)-Ia*	*acm*	1
Unknown	94	12/2011	Urine	GM, AP, CIP, E, CD	*vanA, aac(6′)-Ie-aph(2″)-Ia*	*acm, esp*	3
	95	3/2012	Urine	GM, AP, CIP, E, CD	*vanA*	*acm*	1
	96	5/2012	Urine	GM, AP, CIP, E, CD	*–*	*acm*	8
	97	5/2012	Urine	GM, AP, CIP, E, CD	*vanA, aac(6′)-Ie-aph(2″)-Ia*	*acm*	1
	98	7/2012	Urine	E, CD	*–*	*–*	12
	99	7/2012	Blood	GM, AP, CIP, E, CD	*vanA, aac(6′)-Ie-aph(2″)-Ia*	*acm*	1
	100	8/2012	Urine	AP, CIP, E	*–*	*–*	1
	101	8/2012	Urine	CIP, E, CD	*–*	*acm*	12
	102	8/2012	Urine	AP, CIP, E, CD	*–*	*acm*	15
	103	8/2012	Urine	GM, AP, CIP, E, CD	*vanA, aac(6′)-Ie-aph(2″)-Ia*	*acm*	3
	104	9/2012	Urine	GM, AP, CIP, E, CD	*vanA*	*acm*	16
	105	12/2012	Urine	GM, AP, CIP, E, CD	*vanA, aac(6′)-Ie-aph(2″)-Ia*	*acm, esp, gelE*	3
	106	1/2012	Urine	GM, AP, CIP, E, CD	*vanA, aac(6′)-Ie-aph(2″)-Ia*	*acm, gelE*	1
	107	1/2013	Urine	GM, AP, CIP, E, CD	*–*	*acm, gelE*	24
	108	1/2013	Urine	AP, CIP, E, CD	*–*	*–*	26

^a^*NICU* Neonatal Intensive Care Unit, *CICU* Coronary Intensive Care Unit; *PICU* Paediatric Intensive Care Unit

^b^*m/y* month/year, *CSF* Cerebrospinal fluid

^c^*GM* Gentamicin, *AP* Ampicillin, *CIP* Ciprofloxacin, *E* Erythromycin, *CD* Clindamycin

^d^Inducible resistance to clindamycin

## Discussion

In the current study, the majority (57%) of the isolates was *E. faecium*. This observation is similar to reports from other countries in which the distribution of Enterococcal species derived from clinical samples (blood, urine, pleural fluid, cerebrospinal fluid, sputum, ascites and hydrothorax) was changed in the favour of *E. faecium* [3, 4, 12, 13]. The increase in the prevalence of *E. faecium* species may be due to common resistance of this bacteria to anti-enterococcal drugs, such as ampicillin, aminoglycosides and glycopeptides [3]. In our study, resistance to vancomycin in *E. faecium* and *E. faecalis* isolates was 70 and 9%, relatively. The occurrence of VRE varies in different countries, with a high frequency described in VRE in the US, the UK, Ireland, Saudi Arabia and Turkey [13–16], whereas a low percentage is specific for some European countries such as France and Italy [17, 18]. In spite of past studies in Iran, which showed that all VRE were *vanA* genotype, in our study, this gene was observed in 95 and 50% of VREfm and VREfs [5, 19, 20]. A possible explanation for this variation is probably related to the presence of other resistance gene such as *vanB* or presence of other resistance mechanism including thicker cell wall production [21–23]. Similar to previous finding in Iran, 75% of *E. faecium* and 49% of *E. faecalis* isolates were HLGR [11, 24]. In the current study, 48 and 61% of HLGR in *E. faecium* and *E. faecalis* strains carried the *aac(6′)-Ie-aph(2“)-Ia* gene. This finding was similar with previous studies in which have been shown that the *aac(6’)-Ie-aph(2”)-Ia* gene is the predominant gene responsible for HLGR. [5, 11, 25–27]. In this study, inducible resistance to clindamycin was observed in only 7% of *E. faecium* isolates. Since the resistance to erythromycin and clindamycin antibiotics depends on the use of these agents in the clinic, inducible resistance to clindamycin between *E. faecium* strains might be attributed to the wide intake of these antibiotics in our study center [28]. Our result showed that the *acm* gene was most prevalent virulence gene in *E. faecium* strains. Similar findings were observed in other studies [8, 25, 29]. It seems that the *acm* gene have a role in the improved ability of members of the hospital-associated *E. faecium* to cause disease [30]. Similar to previous report, the prevalence of *ace* and *gelE* genes was 6 and 16% [25]. The *cylA* gene was not detected in any of the 108 *E. faecium* isolates which is in line with the results stated by other investigators who also tested *E. faecium* strains for the presence of *cylA* or more of virulence genes [27, 31]. The rates of *esp* and *asa1* genes were 17.5 and 2%. In some studies, these genes were reported in higher prevalence but in our study and some other studies these genes were identified in lower prevalence among *E. faecium* isolates [32, 33]. Similar to former studies, the presence of the *esp* gene was significant among VRE isolates [34, 35]. Recently, a variant of *esp* gene in VREfm clones has been reported. Also, *esp* gene has been found to be more common in clinical isolates than fecal isolates, which shows the role of *esp* gene in pathogens of enterococci [34, 36]. The MLVA typing of 108 *E. faecium* isolates produced 34 VNTR patterns and 27 MTs. In a study conducted by Top et al. MLVA of 392 *E. faecim* isolates revealed 127 different MTs [9]. In a study piloted by Gawryszewska et al. MLVA of 112 invasive *E. faecium* isolates showed 12 different MTs [3]. Unlike MT1 strains that were isolated from all wards in the 4 years period; two MT2 and MT3 were only found in hospitalized patients in the 4 years of study. Differences in the number of types between the present study and previous studies are probably due to different naming patterns for MTs and the term “VNTR pattern” in the present study is equivalent to MT in two other studies. Three common types (MT1, MT2 and MT3) were resistant to gentamicin, ampicillin, ciprofloxacin, erythromycin, clindamycin and had *acm* and ampicillin resistance, which is more prevalent in nosocomial strains [2], had high frequency in isolates of three common types. This probably indicates the presence of a multi-drug resistant clone that is compatible with the treatment center and the infection control strategies appear to be ineffective so the organism is stable and spreading to patients in different departments and outpatients referring to this center. On the other hand, MT2 and MT3 strains were likely to mutate in order to adapt to the hospital setting. For example the resistance gene pattern *vanA*+ *aac(6′)-Ie-aph(2“)-Ia* and *esp* virulence gene in MT3 were significantly more abundant than MT1. Since the *esp* gene in isolates of *E. faecium* is a marker of a pathogenic island that can be transmitted through conjugation to other isolates and *vanA* and *aac(6’)-Ie-aph(2”)-Ia* genes are often found on plasmids [7, 37], identification of these isolates is necessary in order to review the infection control strategies to prevent the release of resistance genes, *vanA* and *aac(6′)-Ie-aph(2″)-Ia*, and the virulance gene, *esp*, to other cells.

## Conclusions

This study has demonstrated changes over time in species distribution in enterococci isolated from an Iranian children’s hospital. The results show a shift from *E. faecalis* to *E. faecium* as the dominant enterococcal species among patients at the children Hospital. Our data revealed that the majority of *E. faecium* isolates (66%) belonged to three common MTs and these types were isolated from different wards in children hospital. Moreover, the results of this study shows that there is a significant difference in the prevalence rate of antimicrobial resistance and virulence genes among common MTs.

## Methods

### Bacterial isolates

One hundred and eighty-nine non-repetitive isolates of *Enterococcus* spp. were collected during December 2011 to November 2015 from various clinical samples of children admitted to a children hospital in Tehran, Iran. Enterococcal isolates were initially re-identified in the microbiology laboratory of Tehran university of Medical Science based on a series of conventional microbiological tests [38]. To confirm the identity of isolate as *E. faecium* and *E. faecalis,* the *ddl* gene was amplified by a Polymerase Chain reaction (PCR)-based method as described previously [39]. Isolates identified as *E. faecium* were studied further. The study was approved by the Ethics Committee of Tehran University of Medical Sciences.

### Antimicrobial susceptibility testing

Antibiotic susceptibility testing was performed by disc diffusion method according to the Clinical Laboratory Standards Institute (CLSI) guidelines [40] with the following antimicrobial disks (Mast Group Ltd., Merseyside, UK.): ampicillin (10 μg), ciprofloxacin (5 μg), erythromycin (15 μg), clindamycin (2 μg). HLGR isolates were also determined by disk diffusion method by using 120 μg gentamicin disk. Inducible clindamycin resistance was determined by D-test [40]. The minimum inhibitory concentrations (MICs) of vancomycin was determined by the agar dilution method. *E. faecalis* ATCC29212 and *S. aureus* ATCC25923 were used as controls [40].

### Antimicrobial resistance and virulence genes detection

Bacterial genomic DNA was extracted from overnight grown colonies by boiling method [19]. The genes encoding resistance to vancomycin (*vanA*) and aminoglycoside (*aac(6′)-Ie-aph(2″)-Ia*) among *E. faecium* and *E. faecalis* isolates and virulence factor genes (*cylA*, *gelE*, *esp*, *acm*, *ace*, *asa1*) among *E. faecium* were detected by a series of PCR assays [5, 25, 34, 41].

### Molecular analysis

To examine the genotypic diversity of *E. faecium* isolates, MLVA was carried out by a modified Top method [9], as previously described, Briefly, 5 VNTR loci (VNTR-1, VNTR-7, VNTR-8, VNTR-9, VNTR-10) were targeted by PCR using the following steps: an initial denaturation at 95 °C for 5 min and final extension at 72 °C for 5 min. For VNTR-1, 30 cycles of 95 °C for 50 s, 54 °C for 50 s and 72 °C for 80 s were performed. For VNTR-7 a touchdown PCR was done that involved 30 cycles, comprising of 30 s at 95 °C, 30 s at 65 °C down to 55 °C and 1 min at 72 °C. For VNTR-8, VNTR-9, and VNTR-10, 50 s at 95 °C, 45 s at 59 °C and 1 min at 72 °C was prepared. Amplified products were separated by electrophoresis in 2% agarose gels with 0.5X TBE (Tris/Borate/EDTA) buffer. The amplicon bands were visualized with UV illumination after staining with KBC power load dye (GelRed Nucleic Acid Gel Stain, 10,000× in water, Kawsar Biotech Co., Tehran, Iran). MLVA type (MT) were assigned on the basis of one or more loci differences, congruence with a similarity index of approximately 80%. Therefore, MTs were defined as isolates sharing 80% or higher similarity.

### Statistical analysis

The Fisher’s test was used to compare the frequency of antibiotic resistance, virulence factors and resistance genes in common MTs (95% confidence intervals and *P* value ≤ 0.05 considered significant). All results were rounded down if they were < 0.5, were presented as whole numbers if they were > 0.5 and were regarded 0.5 itself if they were = 0.5.

## Data Availability

Please contact author for data requests.

## Abbreviations

Acm: Collagen-binding adhesin
AP: Ampicillin
Asa1: Aggregation substance
CD: Clindamycin
CICU: Coronary Intensive Care Unit
CIP: Ciprofloxacin
CLSI: Clinical and Laboratory Standards Institute
CSF: Cerebrospinal fluid
CylA: Cytolysin
E: Erythromycin
Esp: Enterococcal surface protein
GelE: Gelatinase
GM: Gentamicin
HLGR: High-level gentamicin resistant
m/y: month/year
MIC: Minimal inhibitory concentration
MT_fm_: MLVA Type *E. faecium*
NICU: Neonatal Intensive Care Unit
PCR: Polymerase Chain reaction
PICU: Paediatric Intensive Care Unit
VNTR: Variable number tandem repeat
VRE: Vancomycin resistant enterococci
VREfm: Vancomycin resistant enterococci *E. faecium*
VREfs: Vancomycin resistant enterococci *E. faecalis*
